# Variety-specific selenium accumulation patterns and quality attributes of rice following foliar selenium application

**DOI:** 10.1371/journal.pone.0331517

**Published:** 2025-10-03

**Authors:** Fanbin Meng, Jing Wang, Donghai Wang, Jianfei Wang

**Affiliations:** 1 College of Resource and Environment, Anhui Science and Technology University, Fengyang, China,; 2 Department of Biological and Agricultural Engineering, Kansas State University, Manhattan, Kansas, United States of America; University of Education, PAKISTAN

## Abstract

In the context of addressing regional selenium (Se) deficiencies in China, this study undertook an investigation into the efficacy of foliar Se spray on 12 rice varieties. The primary focus was on assessing rice quality and Se enrichment in various plant components, namely grain, roots, stems, and leaves. The foliar Se spray led to an increase in soil Se content from 0.13 to 0.26 mg. Growth promotion was observed, with rice variety ZLY experiencing a height increase of 19.10 cm, while variety QYX had only a 0.90 cm increase. However, grain weight and numbers were minimally affected. Foliar Se spray had different impacts on rice quality among varieties and treatments. Specifically, variety TLY consistently had the highest numbers of brown and milled rice grains, whereas TXJ and LY had the lowest numbers of brown and milled rice grains respectively. Foliar Se spray had diverse effects on protein contents, resulting in the highest glutelin content of more than 4% and prolamin content as low as less than 0.1%, while the total protein content remained largely unchanged. Moreover, rice varieties demonstrated varying Se enrichment capacities, with the highest for varieties ZLY and QYX grain, and the lowest for LY, TXJ, and JLY grain. The results offered technical support for the selection and promotion of selenium-enriched rice, thereby presenting a potential solution to Se deficiency in China.

## 1. Introduction

Selenium (Se), an essential trace element for human health, is renowned for enhancing immune function and preventing age-related cardiovascular and cerebrovascular diseases [1]. Prolonged deficiency of Se can give rise to various diseases, among which endemic diseases such as Keshan disease are included [2]. According to statistics, over 65% of regions worldwide are at risk of Se deficiency, and in China, the prevalence is approximately 72% [3]. However, the human body cannot synthesize Se and it must be obtained from external sources [4]. Consequently, Se-enriched foods have become a major focus in recent years [5].

Compared with applying Se to the soil, foliar spraying of Se can enhance the utilization of Se fertilizer, reduce Se loss, and to some extent, mitigate soil Se pollution [6]. Foliar spraying of Se has become the main method for Se accumulation in plants [7]. Rice, as a staple food, has a strong capacity for Se bioaccumulation, making it a potential solution to Se deficiency [8]. It should be noted that Se can be a double-edged sword for plants [9]. While optimal Se levels promote plant growth [10], excessive Se can be toxic [11]. Thus, the concentration of foliar spraying of Se has been widely studied [12,13]. And the timing preferences for foliar spraying of Se have also been explored [14]. The effects of different Se forms on rice growth and Se accumulation in roots and grains have also been researched [15]. However, studies on the Se bioaccumulation capacities and its effect on rice quality in diverse rice varieties are scarce.

To explore the effects of Se foliar spray on rice for the bioaccumulation capacities of Se in diverse rice varieties, 12 high-yielding rice varieties grown in Fanshan Town, Lujiang County, Hefei City, Anhui Province, China, have been selected. Simultaneously, the Se enrichment characteristics of different rice varieties and the quality of rice are evaluated. The aim of this study is to obtain Se-enriched rice varieties for large-scale promotion, thereby offering new perspectives for addressing diseases caused by Se deficiency.

## 2. Materials and methods

### 2.1. Experimental area and rice varieties

The field experiments were carried out during the 2021 and 2023 crop season in Fanshan Town, Lujiang County, Hefei City, Anhui Province, China. This location, at an altitude of 486 meters, with a longitude of 117^o^26’5.478” east and a latitude of 31^o^7’10.542” north, is a major rice-producing area. The region’s climate is categorized as subtropical humid monsoon climate, boasting an average temperature of 15.8°C and an average annual rainfall of 1263.2 mm. Twelve locally high-yielding and high-quality rice varieties were selected for the experiment. Details are presented in Table 1.

**Table 1:** Selected rice varieties.

No.	Variety	Type	Abbreviation
1	Ningjing 12	Japonica	NJ
2	Quanyouxiang 66	Indica	QYX
3	Qiuliangyou Xin Zhan	Indica	QLY
4	Xiliangyou 128	Indica	XLY
5	Liangyou 7761	Indica	LY
6	Zhenliangyou 8612	Indica	ZLY
7	Ningxiangjing 9	Japonica	NXJ
8	Tailiangyou 1413	Indica	TLY
9	Jiaheyou 175	Indica	JHY
10	Zhendao 28	Japonica	ZD
11	Taixiangjing 1402	Japonica	TXJ
12	Jiuliangyou 9	Indica	JLY

### 2.2. Materials and methods

The experiment design employed was randomized block with at least three replications [8]. Each variety was allocated an area of 8 meters by 20 meters. Machine-transplanted seedlings were sown on May 10, and machine transplanting was carried out on June 5, with a plant spacing of 25 centimeters by 14 centimeters. Conventional local management practices were adhered to [16]. After harvest, the Agronomic traits including plant height, weight, grain number per panicle, and imperfect grain number were tested in time. Then the polished rice and brown rice were tested. Briefly, the average of six plants in the plot, measured with a graduated ruler from the soil to the tip of the highest panicle to determine plant height (cm). The panicles of 1 m^2^ at six different positions, and six replicates from each treatment, were counted to measure panicle numbers per square meter. The panicles were harvested, threshed manually, and the grains were sun dried and adjusted to ~14% moisture content to determine the yield, grains per panicle and filled grain percentage. Six samples of 1,000 filled grains from each treatment were randomly selected, then weighed to determine the 1,000-kernel weight. The harvested panicles/ears were manually threshed. Grains were visually categorized based on established criteria (e.g., filled vs. unfilled, partially filled, sterile, aborted, chalky) to confirm imperfect grain number.


$$\text{Imperfect grain number}=\frac{\text{Total Florets or Spikelets}}{\text{Panice or Ear}-\text{Nmber of Fully Filled Grains}}\times \text{100}\%$$ (1)

The polished rice were determined according to inspect the milled grains. Where chalky grains, broken grains, undeveloped kernels, and discolored grains were key imperfections shift. The brown rice were determined through visually inspected. Where unfilled/shriveled, partially filled, aborted/sterile, and physically damaged were key imperfections shift.

### 2.3. Se-enriched foliar formula and Se foliar spray protocol

A self-developed Se-enriched foliar fertilizer, with sodium selenite as the main ingredient (chemical pure, 99.5%), was uniformly sprayed on the leaf surfaces during the panicle initiation period [17]. According to the preliminary experimental results of our team, the application amount of the Se-enriched fertilizer was 45 grams per hectare, and its spraying volume was 100 milliliters per square meter.

### 2.4. Analytical procedures

#### 2.4.1. Determination of soil properties.

The content of soil organic matter was determined by the potassium dichromate volumetric method (NYT1121-6-2006) [9]. Available phosphorus in soil was determined by the NaHCO_3_ extraction method [3]. The available potassium in soil was determined by NH_4_OAc extraction and flame photometry [18]. The available nitrogen in soil was determined by the alkaline diffusion method [6]. The soil pH was determined by the potentiometric method [19].

#### 2.4.2. Extraction of protein components.

The rice from different varieties were collected as samples. The extraction method for protein contents was in accordance with the Coomassie Brilliant Blue method [20].

#### 2.4.3. Determination of Se content in rice organs.

The determination of soil and plant Se contents followed the national agricultural standards NY/T 1104-2006 and GB/T 21729-2008 respectively [6]. In brief, ground samples (0.5 g) underwent acid digestion using a mixture of 6 ml HNO_3_ and 2 ml HClO_4_ in a digestion block. The temperature was sequentially increased to 50°C, 100°C, and 150°C, holding each temperature for 60 minutes, and finally raised to 200°C until the extracts became translucent. After digestion, the extracts were quantitatively transferred to volumetric flasks. The final volume was adjusted to 50 ml with deionized water. Se concentrations were measured using inductively coupled plasma optical emission spectroscopy.

#### 2.4.4. Se Increment calculation.

The calculation method for Se increment is given by Equation (1).


$${\text{Se}}_{\text{Increment}}(\%)=\frac{{Se\;Concentrtion}_{with\;foliar\;spray}-{Se\;Concentration}_{witout\;foliar\;spray}}{{Se\;Concentration}_{witout\;foliar\;spray}}$$ (2)

### 2.5. Statistical analysis

Data were analyzed statistically using the IBM SPSS Statistics for Windows (Version 22.0, Armonk, NY, IBM Corp, USA), and graphs were generated using Origin 2021. The overall statistical significance among responses to different treatments was α = 0.05. Multiple comparisons with Tukey’s HSD test were then conducted to identify any significant differences between pairs of treatments. All treatments were conducted in two replicates.

## 3. Results and discussion

### 3.1. Effect of foliar Se spray on soil fertility

Foliar Se spray significantly altered several rice field soil fertility parameters, including total Se, available Potassium, and pH, particularly increasing the total Se content in the soil from 0.13 to 0.26 mg/kg (Table 2). This doubling of soil Se aligns with findings by Chen et al. [15], the increased Se content in the rice field soil was mainly derived directly from foliar Se spray, with a smaller portion released from the rice root system after being absorbed by rice leaves. In other words, some sodium selenite solution, through the conduction of rice leaves, stems, and roots, entered the soil, resulting in a double increase in soil Se content. This is in accordance with refernce [21]. Meanwhile, the treatment also reduced available potassium significantly from 109.20 to 104.07 mg/kg. The decrease in available potassium was possibly due to the potassium’s involvement in the process of Se absorption and transport by rice leaves, leading to the consumption of a small amount of available potassium [4]. This reduction suggests a nutrient trade-off during foliar Se enrichment. Additionally, the pH of rice field soil decreased from 6.02 to 5.64. The decrease in pH was attributed to the reactions between alkaline earth metals and sodium selenite, which can lock part of Ca^2+^ and Mg^2+^, etc., resulting in a decrease in pH [9]. Although Se soil has function to alter soil fertility, it did not show significant effect on organic matter content, total nitrogen, and available phosphorus.

**Table 2:** Effect of Se foliar spray on rice field soil fertility.

Soil Parameter	Total Se (mg/kg)	Organic Matter (g/kg)	Total Nitrogen (g/kg)	Available Phosphorus (mg/kg)	Available Potassium (mg/kg)	pH
Before Se Spray	0.13 ± 0.06a	24.64 ± 0.25a	1.54 ± 0.11a	12.31 ± 0.17a	109.20 ± 0.78a	6.02 ± 0.10a
After Se Spray	0.26 ± 0.08b	25.90 ± 0.21a	1.56 ± 0.13a	12.27 ± 0.19a	104.07 ± 0.22b	5.64 ± 0.12b

### 3.2. Effect of foliar Se spray on agronomic traits and quality and kernel weigh of polished and brow rice

**Fig 1:**
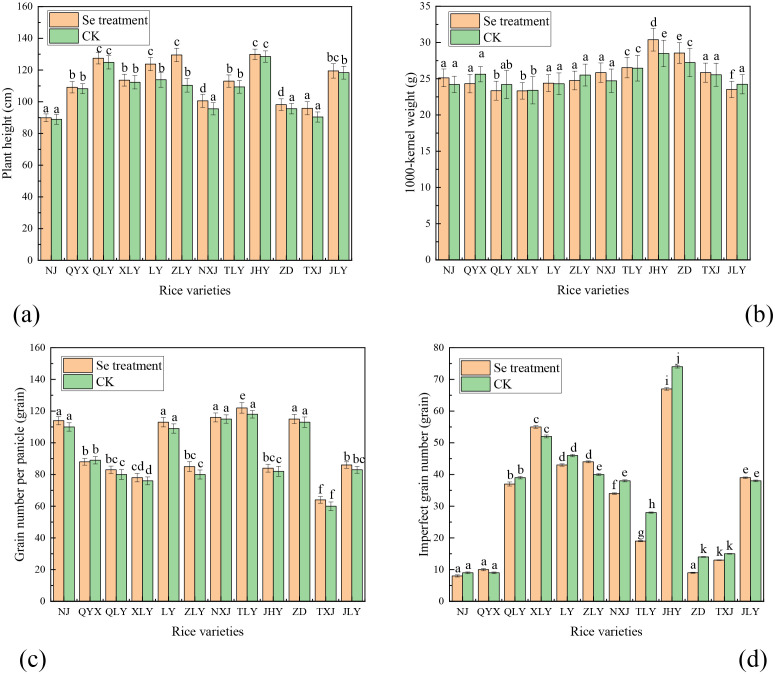
Effect of Se spray on agronomic traits of rice varieties. (a) plant height, (b) 1,000-kernel weight, (c) grain number per panicle, and (d) imperfect grain number.

**Fig 2:**
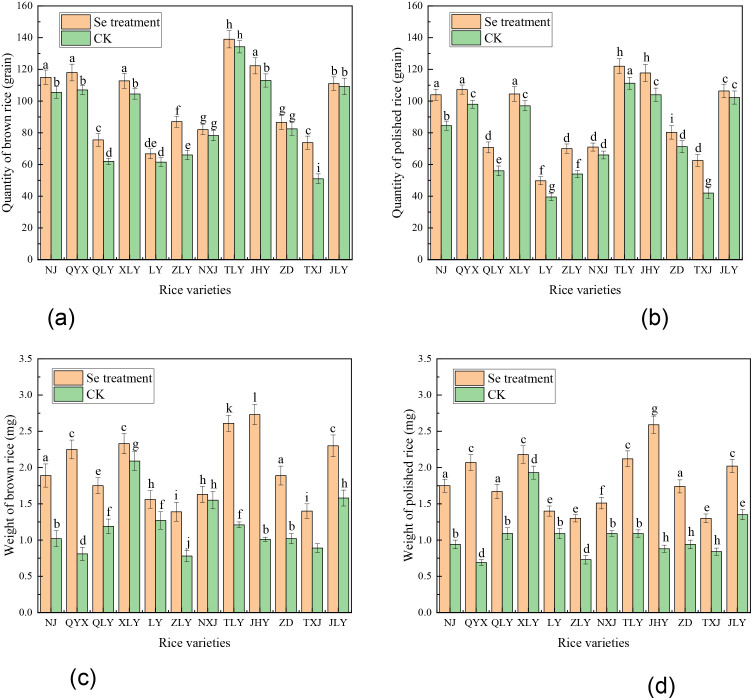
Effect of Se spray on quality and yield of brown and polished rice. (a) quantity of brown rice, (b) quantity of polished rice, (c) weight of brown rice, and (d) weight of polished rice.

Fig 1 shows that foliar Se spray had a significant effect on agronomic traits, including plant height, 1,000-kernel weight, spikelet number, and imperfect grain number, varied with different rice varieties. The foliar Se spray improved plant height across all 12 rice varieties, with ZLY showing the largest gain (19.10 cm) and QYX the smallest (0.90 cm) (Fig 1a). This varietal response supports prior reports by Xu et al. [22] and Di et al. [23] that foliar Se spray treatment can stimulate vegetative growth, though the magnitude of effect remains genotype-dependent. However, the 1,000-kernel weight, grain number per panicle, and imperfect grain number showed irregular trends with little change (Fig 2b–2d), the findings align with Mo et al. [24]. As it is, there are too many factors to affect them. For 1,000-kernel weight, NJ, QYX, TLY, JHY, and ZD had higher weight. For grain number per panicle, NJ, LY, NXJ, TLY, and ZD performed excellently, showing the positive effect on these varieties. For imperfect grain number, NJ, QYX, ZD, and TXJ are all in the range of about 10. The results suggest that NJ and ZD, as two typical varieties, have an advantage for promoting Se-enriched rice.

Fig 2 shows that foliar Se spray enhanced both the quality of rice grain and kernel weight of brown and polished rice, indicating a clear benefit to grain kernel weight and grain quality. These outcomes agree with the observations by Niu et al. [25] and Liu et al. [26,27], increasing the rice kernel weight and rice yield. Among the tested varieties, TLY and JHY exhibited the highest grain kernel weight gains, while TXJ and LY performed the least (Fig 2c, 2d). This variability emphasizes the importance of genetic background in determining response to Se biofortification. Our findings not only validate Se’s agronomic potential for improving grain quality but also identified NJ and JHY as strong candidates for functional rice breeding programs, consistent with prior work by Guo et al. [28]. The results suggest that foliar Se spray has a positive effect on rice quality and kernel weight grain for both brown and polished rice [17].

### 3.3. Effect of foliar Se spray on rice protein content

**Fig 3:**
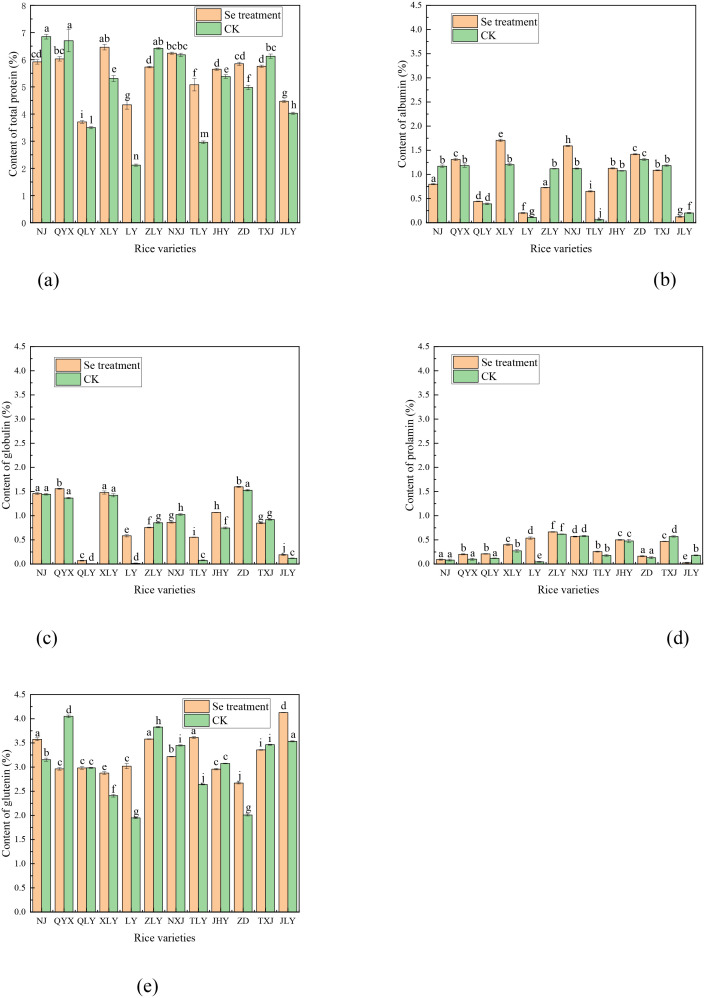
Effect of foliar Se spray on (a) total protein content, and protein fractions. (b) albumin content, (c) globulin content, (d) prolamin content, and (d) glutenin content.

Fig 3 shows that foliar Se spray had significant effects on protein composition (e.g., glutelin, globulin, albumin, prolamin) across varieties, particularly increasing glutelin and globulin contents in most lines. Notably, glutelin remained the dominant protein fraction, exceeding 4% in QYX (control) and JLY (Se-treated grain), consistent with results by Liu et al. [29] and de Oliveira et al. [30]. Albumin and globulin levels varied widely among varieties, revealing genetic specificity in protein response to foliar Se spray treatment. For instance, QYX and XLY accumulated higher albumin, NJ and ZD showed increased globulin content, and the varieties LY (control) and JLY (Se treatments) showed lowest albumin content (<1%). In addition, varieties LY and JLY had relatively lower albumin protein content in grain, whereas QYX, XLY, and ZD had relatively higher clear protein content. Similarly, varieties QLY and JLY showed comparatively lower globulin content, while NJ, XLY, and ZD had higher levels. These differences align with reports by Liu et al. [29], who noted selenium’s role in regulating protein expression. Although total protein content remained relatively stable, the observed shifts in protein fractions suggest that Se biofortification can tailor rice nutritional profiles through selective variety use [30]. Therefore, by choosing suitable rice varieties, a pathway for targeted protein production through selenium treatment has been discovered [10].

### 3.4. Distribution of foliar Se spray in different rice organs

**Fig 4:**
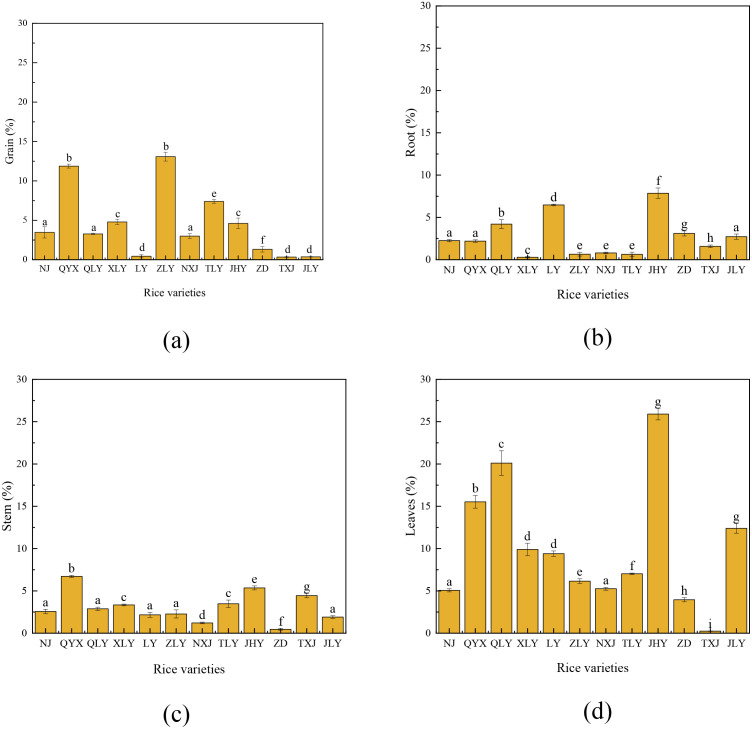
Se accumulation in rice varieties. (a) grain, (b) root, (c) stem, and (d) leaves.

Fig 4 shows that there are notable differences in Se accumulation among various plant parts including grain, roots, stems, and leaves, among the different varieties. The highest Se concentrations were found in the grains of ZLY and QYX, roots of JHY and LY, stems of QYX and JHY, and leaves of JHY. Conversely, the lowest Se concentrations were in the grains of LY, TXJ, and JLY, roots of XLY, ZLY, NXJ, and TLY, stem of ZD, and leave of TXJ. Based on Se accumulation capability, varieties ZLY and QYX can be cultivated to obtain Se-rich grain; LY and JHY are the good candidates to serve as phytoremediators for Se-enriched roots; QYX, JHY, and QLY are preferred to be used as fodder to address Se deficiency in grazing animals such as cattle and sheep. Our results confirm the findings of Wu et al. [31], who observed similar Se localization patterns, as well as that Lavu et al. [32] reported that the Se enrichment capacity can be ranked by the order of leaves, stem, grain, and root. Among all plant parts, leaves consistently exhibited the highest Se enrichment capacity, likely due to its direct contact with the high Se concentration during foliar Se spray [33]. Additionally, Se’s differential distribution among the tissues also highlights its synergistic role in photosynthetic enhancement, leading to strong Se fixation in the leaves, supporting the findings of Ding et al. [7]. Furthermore, Se-enriched grain may be due to the inherently stronger expression of Se accumulation genes and greater stress resistance in naturally Se-rich rice [12]. This underscores that different rice varieties possess varying Se enrichment capacities, and the Se enrichment abilities of different rice plant parts also vary. The research findings advance our understanding of Se transport mechanisms in rice and offer practical guidelines for breeding Se-enriched crops tailored to specific nutritional or environmental applications.

## 4. Conclusions

Foliar Se spray exhibited diverse effects on rice field soil fertility, plant growth, grain quality, and protein composition across different rice varieties. It significantly increased soil Se content and the Se content in the soil is nearly doubled. It promoted the plant growth with limited impact on grain weight and yield. The Se enrichment capacity differed among plant parts and rice varieties. While foliar Se spray had minimal impact on total protein content, it influenced the composition of specific proteins, and its effect on grain quality varied by variety. These research findings demonstrated that selenium treatment has a significant effect on rice quality and underscore varietal differences in Se accumulation. This study provides valuable guidance for selecting selenium-enriched rice for various applications. For future research, it is recommended to investigate the effects of foliar Se spray on additional nutritional attributes (e.g., antioxidants, minerals, vitamins), explore varying application conditions (e.g., different concentrations, timing, and Se compounds), account for broader environmental variability, assess long-term impacts, and evaluate consumer acceptability.

## Supporting information

S1 File Detailed information of the Tables 1, 2 and Figs 1–4. (XLSX)
